# Functional Connectivity in Multiple Cortical Networks Is Associated with Performance Across Cognitive Domains in Older Adults

**DOI:** 10.1089/brain.2014.0327

**Published:** 2015-10-01

**Authors:** Emily E. Shaw, Aaron P. Schultz, Reisa A. Sperling, Trey Hedden

**Affiliations:** ^1^ Athinoula A. Martinos Center for Biomedical Imaging, Department of Radiology, Massachusetts General Hospital, Charlestown, Massachusetts.; ^2^ Department of Psychiatry, Massachusetts General Hospital, Harvard Medical School, Boston, Massachusetts.; ^3^ Department of Neurology, Massachusetts General Hospital, Harvard Medical School, Boston, Massachusetts.; ^4^ Department of Neurology, Center for Alzheimer Research and Treatment, Brigham and Women's Hospital, Harvard Medical School, Boston, Massachusetts.; ^5^ Department of Radiology, Massachusetts General Hospital, Harvard Medical School, Boston, Massachusetts.

**Keywords:** cognition, cognitive control, default mode network, memory, resting-state functional connectivity MRI

## Abstract

Intrinsic functional connectivity MRI has become a widely used tool for measuring integrity in large-scale cortical networks. This study examined multiple cortical networks using Template-Based Rotation (TBR), a method that applies *a priori* network and nuisance component templates defined from an independent dataset to test datasets of interest. *A priori* templates were applied to a test dataset of 276 older adults (ages 65–90) from the Harvard Aging Brain Study to examine the relationship between multiple large-scale cortical networks and cognition. Factor scores derived from neuropsychological tests represented processing speed, executive function, and episodic memory. Resting-state BOLD data were acquired in two 6-min acquisitions on a 3-Tesla scanner and processed with TBR to extract individual-level metrics of network connectivity in multiple cortical networks. All results controlled for data quality metrics, including motion. Connectivity in multiple large-scale cortical networks was positively related to all cognitive domains, with a composite measure of general connectivity positively associated with general cognitive performance. Controlling for the correlations between networks, the frontoparietal control network (FPCN) and executive function demonstrated the only significant association, suggesting specificity in this relationship. Further analyses found that the FPCN mediated the relationships of the other networks with cognition, suggesting that this network may play a central role in understanding individual variation in cognition during aging.

## Introduction

During the course of the life span, older individuals exhibit decreased performance across a range of cognitive domains, including processing speed, executive functions, and episodic memory (Park et al., 2002; Salthouse, 2011; Schaie, 1996; Verhaeghen and Salthouse, 1997). Although these cognitive alterations cut across many domains, alterations in different domains may result from underlying brain networks that are differentially impacted during the course of aging (Buckner, 2004; Hedden and Gabrieli, 2004; Jagust, 2013). For example, we have hypothesized that alterations in the frontoparietal control network (FPCN) are linked to executive function deficits and likely arise from different neurodegenerative processes than those impacting the default network (DN), hypothesized to be linked to episodic memory (Buckner, 2004; Hedden and Gabrieli, 2004; Hedden et al., 2009, 2012a, 2012b, 2014). Functional connectivity MRI (fc-MRI) provides a tool for the investigation of multiple intrinsic brain networks by detecting spontaneous correlations between fluctuations in regional brain activity while a subject rests passively in the scanner, and provides measures of network topography and integrity (Biswal et al., 1995; Van Dijk et al., 2010). In this study, we investigated how functional connectivity in multiple cortical networks was related to performance across multiple cognitive domains in a group of clinically normal older adults.

Subjects with neurodegenerative diseases have reduced network correlations compared to healthy controls (Greicius et al., 2004; Seeley et al., 2009), and healthy older adults have reduced correlations across multiple networks compared to healthy younger adults (Andrews-Hanna et al., 2007; Geerligs et al., 2014). However, examinations of how aging impacts the relationship between alterations in network connectivity and cognition have been inconsistent in the cognitive tasks examined and the methods for defining network connectivity or have been based on relatively small sample sizes (see Ferreira and Busatto, 2013, for review). In a study examining multiple cognitive domains, Andrews-Hanna et al. (2007) found that correlations between the medial prefrontal cortex and posterior cingulate/retrosplenial cortex were positively related to composite scores of processing speed, executive functioning, and episodic memory among older adults. While these results suggest that individual differences in cognition among healthy older adults are related to network connectivity, they did not examine relationships with cognition across multiple networks to determine the specificity or generality of such associations.

In this study, we apply a recently developed functional connectivity method and relate individual estimates of connectivity in multiple cortical networks to performance in multiple cognitive domains. We measured network connectivity using the Template-Based Rotation (TBR) method (Schultz et al., 2014), in which cortical network parcellations are defined from a reference data set and applied as templates to a target data set. The selected parcellation [described in Schultz and colleagues (2014)] provides a set of network templates that include the major networks previously identified from resting-state and task-based studies as having important cognitive roles (Damoiseaux et al., 2006; Laird et al., 2011; Menon and Uddin, 2010).

On an *a priori* theoretical basis, we examined only those network templates corresponding to four cortical networks hypothesized to be associated with the available cognitive measures: the DN, the FPCN, the salience network (SN), and the dorsal attention network (DAN). Although naming schemas have varied across studies, networks corresponding to the spatial pattern of these four cortical templates have been represented in multiple, independent, and large-sample parcellations (Biswal et al., 2010; Smith et al., 2009; Yeo et al., 2011).

Cognition was assessed using *a priori* factor scores (Hedden et al., 2012a) of processing speed, executive function, and episodic memory. Based on prior theoretical and empirical work (Wang et al., 2010), we hypothesized that the DN would have a preferential positive relationship with memory; however, based on the results of Andrews-Hanna and colleagues (2007), we examined the alternative hypothesis of a positive correlation between the DN and all cognitive domains. We also hypothesized a preferential positive relationship between the FPCN and executive function, based on prior data that relate structural measures and functional activity in regions within this network to executive function (Gordon et al., 2015; Hedden and Gabrieli, 2010; Hedden et al., 2012b). Because connectivity in the SN has been linked to motivation and attention (Ham et al., 2013; Onoda et al., 2012; Seeley et al., 2007), we hypothesized nonspecific positive relationships across cognitive domains. Similarly, the DAN has been implicated in attention, memory, and executive functions (van den Heuvel and Hulshoff Pol, 2010); hence, we expected nonspecific positive relationships across cognitive domains. Based on results indicating that the FPCN is intrinsically connected to and flexibly aligns with other networks depending upon task state (Spreng et al., 2010, 2013), which may indicate that the FPCN coordinates control of these other networks (Cole et al., 2012, 2013), we additionally examined the possibility that relationships of the DN, SN, and DAN to cognition were mediated by the FPCN.

## Materials and Methods

### Sample characteristics

Participants were 276 (159 female) cognitively normal, community-dwelling older adults [65–90, *M*=74.0, standard deviation (SD)=6.0] participating in the Harvard Aging Brain Study. Included subjects had completed both baseline neuropsychological testing and an MR scan session. Participants were generally well educated (years of education: *M*=15.8, SD=3.1), had high estimated verbal intelligence quotient (VIQ: *M*=120.8, SD=9.3 estimated from the American National Adult Reading Test) (Ryan and Paolo, 1992), and high socioeconomic status (*M*=28.0, SD=14.7; the scale ranges from 11 to 77 with lower scores indicating higher SES) (Hollingshead, 1957). All participants had a Clinical Dementia Rating of 0 (Morris, 1993), performed no worse than 1.5 SD units below the age- and education-corrected normative score for Logical Memory IIa, a subtest of the Wechsler Memory Scale-Revised (Wechsler, 1987), and scored 26 or above on the Mini-Mental State Examination (Folstein et al., 1975). Participants were also excluded if previously diagnosed with a neurological or psychiatric condition, or if they scored greater than 11 on the Geriatric Depression Scale (Yesavage et al., 1983). The participants in this analysis are a superset of the Harvard Aging Brain sample reported in other analyses involving cognition or functional connectivity (Amariglio et al., 2012a, 2012b; Hedden et al., 2012a, 2014; Huijbers et al., 2014; Mormino et al., 2014; Rentz et al., 2011; Schultz et al., 2014; Sepulcre et al., 2013). All participants had normal or corrected to normal vision and provided informed consent in accordance with protocols approved by the Partners Healthcare, Inc. Institutional Review Board.

### Neuropsychological factors

Factor scores for the cognitive abilities of processing speed, executive function (a second-order factor composed of subdomains of fluency, working memory, and switching), and episodic memory were examined based on a previously published confirmatory factor analysis (Hedden et al., 2012a). Neuropsychological tests included in that analysis were phonemic and category fluency, Letter-Number Sequencing, Digit Span Backward, Self-Ordered Pointing, the Number-Letter task, a modified Flanker task, the Trail Making test, the Digit-Symbol test, the Face-Name Associative Memory Examination, the Six-Trial Selective Reminding test, and the Memory Capacity test. A description of measures used from each neuropsychological test and the derivation of factor scores from a subset of the current sample have been previously published (Hedden et al., 2012a). As in the prior report, all neuropsychological tests were scaled such that higher scores indicated better performance (e.g., faster reaction times were associated with higher processing speed factor scores). To allow comparability across reports and to ensure *a priori* testing of relationships between cognition and functional connectivity, the factor weightings from that prior report were used to compute cognitive factor scores for processing speed, executive function, and episodic memory. A comparison of the factor weightings in the additional participants from the current sample to the prior sample found convergence in the factor structure across samples. Factor scores were z-transformed before analysis. Due to missing data, factor scores for one or more cognitive domains were not computed for four subjects (two missing processing speed, one missing executive function, and two missing episodic memory). Because of our *a priori* research focus on the cognitive abilities of processing speed, executive function, and episodic memory (Andrews-Hanna et al., 2007; Hedden et al., 2012b, 2014) and our approach to limiting the test-specific variance through the use of aggregate factor scores (Salthouse, 2011), specific neuropsychological tests and subdomains of executive function were not examined to limit the number of tests conducted.

### MRI acquisition

Data for functional connectivity analysis were acquired as part of a larger protocol (Hedden et al., 2012a, 2014) on a Siemens TrioTim 3.0 Tesla scanner (Siemens, Erlangen, Germany) paired with a 12-channel phased-array head coil. A gradient-echo echo-planar pulse sequence sensitive to blood oxygenation level-dependent contrast was acquired using the following parameters: TR=3000 msec, TE=30 msec, flip angle=85°, 3.0×3.0×3.0 mm voxels. Forty-seven interleaved transverse slices aligned to the anterior/posterior commissure plane covered the whole brain and were acquired for 124 time points in each of the two runs. Participants were instructed to lie still and remain awake with eyes open during each run. All resting data were acquired before any task acquisitions.

### Data preprocessing

The first four time points of each run were discarded to allow for T1-equilibration effects. Resting-state data were processed using SPM8 (www.fil.ion.ucl.ac.uk/spm/; version r4290). Each run was slice-time corrected, realigned to the first volume of each run with INRIAlign (www-sop.inria.fr/epidaure/software/INRIAlign/) (Freire and Mangin, 2001), normalized to the MNI 152 EPI template (Montreal Neurological Institute, Montreal, Canada), and smoothed with a 6-mm FWHM Gaussian kernel. Following these standard preprocessing steps, additional processing known to be beneficial for fc-MRI analysis was conducted, including (sequentially and in this order) (1) regression of realignment parameters (plus first derivatives) to reduce movement artifacts on connectivity and (2) temporal band-pass filtering (second-order Butterworth filter) to focus the analysis on frequencies in the 0.01–0.08 Hz band. Runs were discarded from further analysis if any one of the following quality assessment (QA) conditions were met: lower than a threshold of 115 for slice-based signal-to-noise ratio (SNR), higher than a threshold of 0.2 mm for mean movement, or more than 20 outlier volumes (defined as a change in the global signal >2.5 SD attributable to the volume, or a change in position >0.75 mm or rotation >1.5° from the previous volume). Across all participants, 17 (3.1%) total runs were discarded; 15 of these included a failure attributable to excessive movement. Outlier volumes (as defined using the above parameters) were not explicitly censored; if censored, the resulting data have a correlation >0.99 with the reported data for all examined networks (this correlation will vary with the definition of outlier volumes).

### Template-Based Rotation

Functional connectivity estimates were derived using the TBR method [detailed in Schultz and colleagues (2014)]. Briefly, this method maps the variance in each functional run onto a set of network templates derived from a reference dataset [here, the 675 participant dataset described in Schultz and colleagues (2014), resulting in 20 component templates, including global and noise components]. The TBR method is conceptually similar to the dual-regression independent component analysis (ICA), but differs in the details of implementation [as described in Schultz and colleagues (2014), with mathematical derivation]. Each template uses information from all voxels in the brain and represents a spatial pattern present in the reference dataset, similar to a component derived from ICA (in fact, ICA components could be used as templates in a TBR analysis). The TBR method effectively regresses a matrix representing all voxels at each time point for an individual subject onto the matrix of all voxels for each template in the reference dataset. For each subject, the resulting beta-estimates for each template reflect the unique variance attributable to a time point in the subject's data and are interpretable as a timecourse that can be used to create standard correlation-based maps of connectivity (one map per template). Although each such map will encompass every voxel in the brain, we extracted connectivity estimates only from a subset of voxels above a threshold in the reference dataset. The TBR method has the advantage of allowing computation of individual estimates of connectivity within a set of networks whose topography has been defined in advance on a reference dataset. One assumption of TBR is that the reference templates are sufficient exemplars of each network to recover sensible measurements of the spatial pattern and connectivity strength within each network of interest. To the extent that an individual's network topography is substantially different from a template, this individual is likely to exhibit reduced connectivity. Comparisons to ICA and seed-based approaches have been previously published (Schultz et al., 2014).

**FIG. 1.**
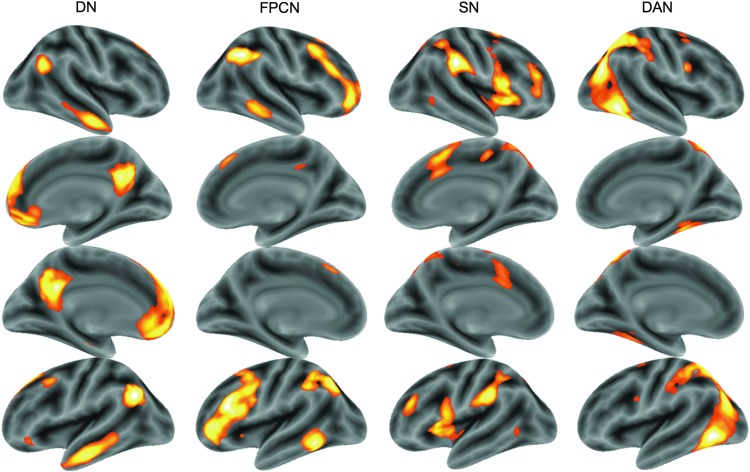
Template maps for the cortical networks of interest. Network templates were defined from an independent dataset and probed in the current dataset for relationships to cognition. Right and left surface renderings, as defined in the reference dataset, are shown for the default network (DN), frontoparietal control network (FPCN), salience network (SN), and dorsal attention network (DAN). Color intensities indicate factor loading of each voxel with the network template in the reference dataset. Color images available online at www.liebertpub.com/brain

On an *a priori* theoretical basis, we examined only those network templates corresponding to four cortical networks hypothesized to be associated with cognition: the DN, FPCN, SN, and DAN (Fig. 1). In the reference dataset, the FPCN is represented by two templates, consisting of the left and right hemisphere regions of the FPCN. We averaged the resulting estimates from these two FPCN templates to compute a single estimate of FPCN connectivity. In the reference dataset, the DN and SN are represented in the same template, with positive factor loadings associated with the DN and negative factor loadings associated with the SN. For present purposes, we split this template into two networks and reverse scaled the SN, so that positive values indicated a greater association with the SN. Notably, since no global signal regression was performed, this relationship was not induced. Connectivity estimates were derived for the DN, FPCN, SN, and DAN corresponding to the average correlation of all voxels identified as associated with that network in the reference dataset (defined as all voxels ≥40% of the maximum factor loading in each template, as shown in Fig. 1); this measure represents an individual's overall connectivity within a network template. To account for remaining differences due to data quality, functional connectivity estimates from each network were corrected for associations with the across-run average for SNR, mean movement, and number of outlier volumes (collectively referred to as QA metrics) through regression before entry into the statistical models.

### Network and cognition factor scores

In addition to the network-specific measures derived from TBR and the domain-specific cognitive factor scores, we examined the potential for general relationships across networks and cognitive domains by computing the commonalities between networks or between cognitive domains. Using principal components analysis, we extracted the first principal component of the four network measures (DN, FPCN, SN, and DAN). This component accounted for 73% of the variance in the network measures, and the correlations between the component and each network were DN=0.94, FPCN=0.87, SN=0.91, and DAN=0.69. Within the older adults, the resulting network component was correlated with age (*r*=−0.24, *p*<0.001) and with the QA metrics (*p*<0.001). Similarly, we extracted the first principal component of the three cognitive domains. This component accounted for 80% of the variance in the cognitive measures, and the correlations between the component and each domain were processing speed=0.90, executive function=0.95, and episodic memory=0.83. The resulting domain-general component was correlated with age (*r*=−0.30, *p*<0.001).

### Correlation analyses

Pearson correlation and partial correlation analyses were conducted in SPSS v21 (IBM, Armonk, NY) to compare each cortical network with each cognitive variable. In hypothesis-driven analyses, we applied a threshold of *p*<0.05, one-tailed with a false discovery rate (FDR) correction for multiple comparisons using the Benjamini–Hochberg procedure (Benjamini and Hochberg, 1995). All analyses controlled for age and QA metrics (see Supplementary Table S1 for unadjusted correlations; Supplementary Data are available online at www.liebertpub.com/brain).

### Voxelwise correlation analyses

The regional topography of voxel-level relationships between network connectivity and cognition was examined using the general linear model implemented in GLM Flex (http://mrtools.mgh.harvard.edu/index.php/GLM_Flex) and visualized using the Functional Image Visualization Environment (FIVE; http://mrtools.mgh.harvard.edu/index.php/Downloads). Each subject's map of network connectivity (representing the variance in each voxel associated with the timecourse for the specified network template) was entered into an analysis with the cognitive variable of interest, age, and QA metrics as regressors.

For analyses involving specificity between each network's connectivity and each cognitive domain, additional covariates representing mean connectivity in the other networks were added to the model. For example, in analyses involving FPCN connectivity and executive function, covariates representing mean connectivity in the DN, SN, and DAN were added to the model. For these first-order regression analyses with covariates, results from GLM Flex will be identical to those produced by SPM8 (www.fil.ion.ucl.ac.uk/spm/software/spm8). Each analysis was restricted to those voxels included in the network of interest as identified in the reference dataset (as defined above and shown in Fig. 1) and thresholded for descriptive purposes at *p*<0.05 (corresponding to *r*≈0.10) with a cluster size=50 or at *p*<0.001 (corresponding to *r*≈0.19) with no minimum cluster size. The lower threshold was purposefully chosen to be liberal (but with a relatively high associated cluster extent), given that we had no regional hypotheses about the pattern of correlations within a network and prefer to err in favor of showing potential regions of interest for future investigation. The upper threshold (*p*<0.001) was chosen to enable a constant threshold across contrasts (as corrected measurements will vary depending upon the network examined); for most comparisons, this threshold was more conservative than an FDR correction.

### Mediation models

To examine the potential mediating role of the FPCN on the relationships between the other networks and cognition, we examined mediation and moderation models using the PROCESS SPSS macro (using the Model 4 and Model 1 templates, respectively), which estimates path effects using ordinary least-squares regression (Hayes, 2013). The significance of direct and indirect effects was assessed with a 95% confidence interval, corresponding to *p*<0.05 two-tailed, using 10,000 bootstrap iterations and accepted if the interval did not overlap zero. Standardized coefficients (achieved by z-scoring all variables before entry in the model) are reported to aid in comparison across models.

## Results

### Network connectivity relationships to cognition

Because age and the QA metrics were significantly related to the network connectivity measures (Supplementary Table S1), we examined the relationships between cortical network connectivity and cognition while controlling for age and QA metrics. The observed relationships between cortical networks and cognitive factor scores remained significant after correction for multiple comparisons (Table 1). The DN and the FPCN had significant positive correlations with all three cognitive domains, and the SN had a significant positive correlation with processing speed and episodic memory (*p*≤0.007). No significant relationships between the DAN and cognition were observed. To examine the degree to which these relationships were general across networks and cognitive domains, we examined the partial correlation of the first principal component from a factor analysis of the networks and, separately, from a factor analysis of the cognitive domains, controlling for age and QA metrics. This correlation was *r*=0.22, *p*<0.001, suggesting a common relationship of network connectivity to general cognition.

**Table 1.** Correlations Between Cognition and Cortical Network Connectivity, Controlling for Age and Quality Assurance Metrics

	*Processing speed*		*Executive function*		*Episodic memory*	
	r	p	r	p	r	p
DN	**0.21**	<0.001	**0.17**	0.003	**0.17**	0.003
FPCN	**0.24**	<0.001	**0.27**	<0.001	**0.21**	<0.001
SN	**0.15**	0.006	0.11	0.034	**0.15**	0.006
DAN	0.09	0.067	0.09	0.080	0.02	0.370

Correlations between cognitive factor scores and cortical networks within older adults controlling for age, signal-to-noise ratio, movement, and number of outlier volumes. Bold values indicate significance at *p*<0.05, one-tailed after false discovery rate correction for multiple comparisons.

DAN, dorsal attention network; DN, default network; FPCN, frontoparietal control network; SN, salience network.

### Topography of network connectivity relations to cognition

**FIG. 2.**
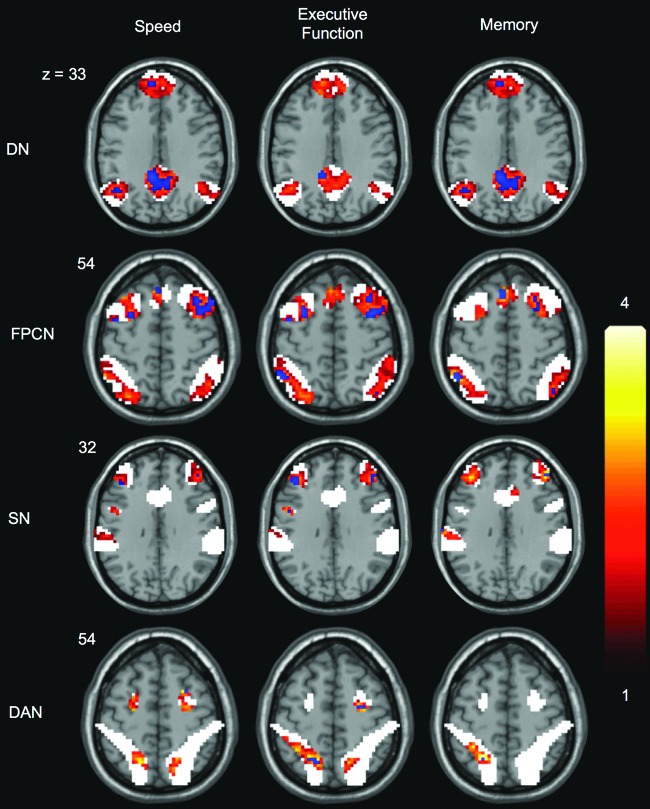
Voxelwise correlations with cognition. Correlations were restricted to voxels within the mask of each network template (white). Representative slices showing that the largest regional correlations are displayed for each combination of network and cognitive domain. Each row is displayed at the same z-coordinate indicated at the left. Correlations were corrected for age and quality assessment metrics. The red-yellow spectrum indicates results exceeding a liberal threshold of *p*<0.05, cluster extent=50. The blue regions exceeded a more conservative threshold of *p*<0.001. Color images available online at www.liebertpub.com/brain

The above analyses indicate that network-wide measures of connectivity are related to cognition. To examine the potential for regional differences in topography of relationships with cognition, we explored the voxelwise correlations of connectivity with each cognitive domain. Because we did not hypothesize specific regional relationships to cognition, these results should be taken as descriptive in nature and are shown primarily to spur novel hypotheses. To reduce the potential for false positives from voxels having a very low association with each network, these analyses were restricted to the voxels identified as highly associated with each network template in the reference dataset (Fig. 1). Above-threshold voxels in these analyses reflect significant correlations between cognition and the connectivity of that voxel with the network as a whole (Fig. 2). Of note, although the network-wide measures in the analyses above indicated associations with cognition, the voxel-level results demonstrated substantial regional variation in the strength of the correlation between network connectivity at a given voxel and each cognitive domain. These results, presented at a liberal threshold, suggest that relationships with cognition likely occur across multiple regions within a network and are general across cognitive domains, but that there may nonetheless be important regional variability to be explored. In particular, although the SN and DAN had weak or no relationships with cognition when using network-wide metrics (Table 1), these networks showed evidence of regionally specific relationships with similar magnitude to those observed in the DN and FPCN (Fig. 2).

### Specificity of network relations to cognition

**FIG. 3.**
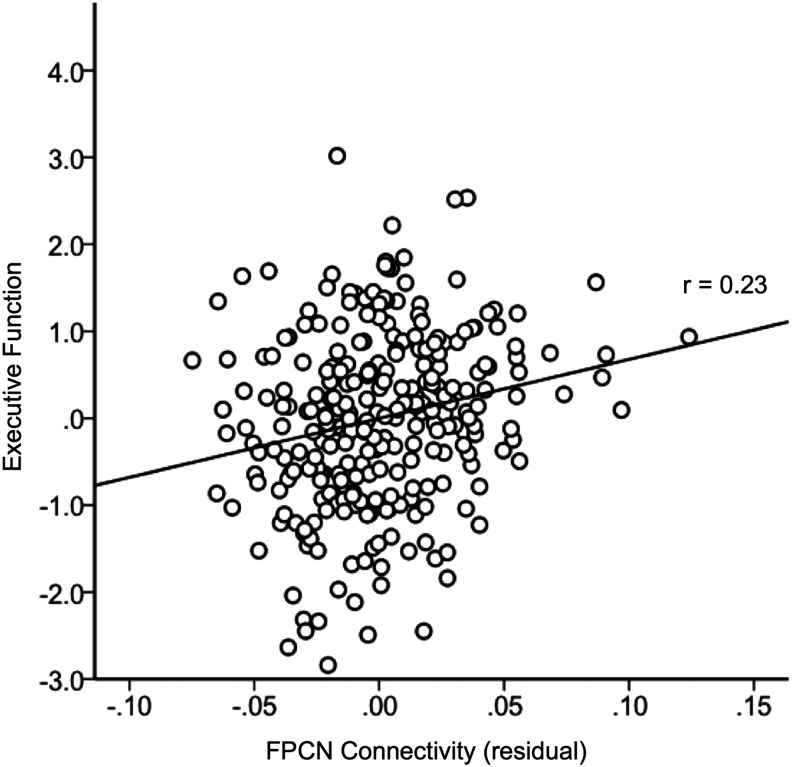
Partial correlation of FPCN connectivity with executive function. Scatterplot is shown after residualizing FPCN connectivity on age, quality assessment metrics, and average connectivity in the other three networks.

One feature of the TBR method is that the network templates are not required to be orthogonal to one another, and each voxel will be assigned a value representing its association to every template in the analysis. Because the resulting network connectivity metrics are correlated with each other (Supplementary Table S1), it remains possible that the above results represent a general influence of an individual's average connectivity across networks on cognition, rather than a specific relationship between connectivity in a given network and a particular cognitive domain. We therefore examined the correlations between connectivity in each network and each cognitive domain controlling for age, QA metrics, and connectivity in each of the other three networks. Because the DN and SN were highly correlated, we conducted analyses both including and excluding these networks as covariates for one another; results did not substantially differ. This analysis revealed that the only significant relationship exhibiting specificity was between the FPCN and executive function (Fig. 3; *r*=0.23, *p*<0.001, one tailed, FDR corrected). Notably, there was no specific association between the DN and episodic memory when controlling for the other three networks (*r*=0.04, *p*=0.29, one tailed, uncorrected).

**FIG. 4.**
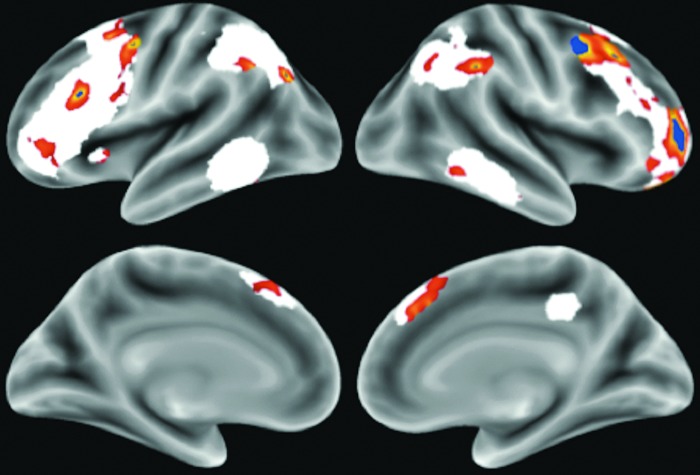
Voxelwise partial correlations of the FPCN with executive function. Correlations were restricted to voxels within the mask of the left and right FPCN templates (white) and displayed on an inflated surface map. Correlations were corrected for age, quality assessment metrics, and average connectivity in the other three networks. The red-yellow spectrum indicates results exceeding a liberal threshold of *p*<0.05. The blue regions exceeded a more conservative threshold of *p*<0.001. Color images available online at www.liebertpub.com/brain

To explore the topography of this specificity, we repeated the voxelwise correlation between FPCN connectivity and executive function, while simultaneously controlling for average connectivity in the DN, SN, and DAN (Fig. 4). Again, we did not have regionally specific hypotheses and these results are presented for descriptive purposes. This analysis was conducted separately for the left and right FPCN templates. As expected, the results were reflective of, but less robust than, the nonspecific relationships described above. The largest clusters of regional correlations with executive function were observed in the left and right dorsolateral prefrontal cortex, with smaller above-threshold clusters observed in the presupplementary motor area and in the bilateral parietal lobule. Notably, it appears there are at least three different loci in the dorsolateral prefrontal cortex, suggesting regional segregation within this larger area that is consistent with a previous functional parcellation (Wig et al., 2014).

### Mediating relationships among networks

**FIG. 5.**
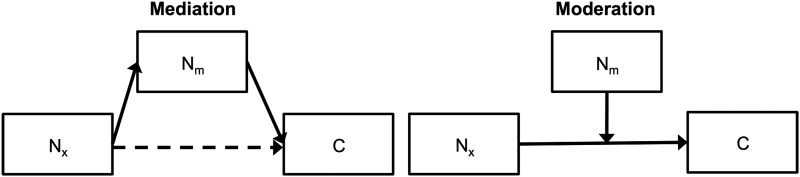
Mediation and moderation models of network connectivity to cognition. In a mediation relationship (left), the effect of a network's connectivity (N_x_) on cognition (C) acts through a mediator network's connectivity (N_m_). For full mediation to be supported, the direct effect of N_x_ on C is expected to no longer be significant after controlling for the mediating role of N_m_, as indicated by the dashed path. In a moderating relationship (right), the effect of a network's connectivity (N_x_) on cognition (C) is altered in the presence of a moderating network (N_m_). Moderation is tested by examining the interaction effect of N_x_ and N_m_ on C.

Because of its potential role as an intermediary for communication between networks and for the output of network processing to behavioral effectors, we examined the FPCN as a mediator of the relationships of the DN or SN to cognition (Fig. 5, left, and Table 2). The DN had significant direct relationships to all measures of cognition, and the FPCN had a significant mediating influence (indicated by a significant indirect effect, N_x_−N_m_−C) on this relationship for all cognitive variables except memory. Although the indirect effect through the FPCN was not significant for memory, neither was the remaining direct effect of the DN on memory significant (N_x_′−C), suggesting that the relationship of the DN to memory was reduced by the inclusion of the FPCN in the model. For the SN, the FPCN had a significant indirect influence on all relationships to cognition. To ensure that the mediating influence of the FPCN was specific, rather than a general influence of one network to any other, we also examined models in which the FPCN was the exogenous (N_x_) variable and the DN or SN was the mediating (N_m_) variable. No such models exhibited a significant indirect effect. To determine whether the effect was a mediating or moderating influence, we additionally examined moderation effects of the FPCN on the DN or SN relationships to cognition (Fig. 5, right). No such models exhibited a significant interaction effect. Because the DAN had no significant direct relationships to cognition, these models did not meet the criteria for potential mediation and are therefore not reported in detail; we note, however, that the indirect path from the DAN to FPCN to cognition was significant for all cognitive domains.

**Table 2.** Mediation Models

*C*	*N_x_−C*	*N_x_−N_m_*	*N_m_−C*	*N_x_−N_m_−C*	*N_x_′−C*
N_x_=DN, N_m_=FPCN					
Speed	0.19^a^	0.70^a^	0.17^a^	0.12^a^	0.07
Executive function	0.15^a^	0.70^a^	0.29^a^	0.20^a^	−0.05
Memory	0.16^a^	0.70^a^	0.16	0.11	0.05
Global	0.19^a^	0.70^a^	0.23^a^	0.16^a^	0.03
N_x_=SN, N_m_=FPCN					
Speed	0.14^a^	0.62^a^	0.22^a^	0.13^a^	0.01
Executive function	0.10	0.62^a^	0.31^a^	0.19^a^	−0.09
Memory	0.14^a^	0.62^a^	0.17^a^	0.10^a^	0.04
Global	0.15^a^	0.62^a^	0.26^a^	0.16^a^	−0.01

Standardized path coefficients for mediation models involving FPCN as a mediator of the network to cognition relationships for the DN and SN. Refer to Figure 5 (left) for model structure. Network measures were controlled for age and quality assessment metrics before entry in the models. N_x_−N_m_−C indicates the indirect (mediated) effect of N_x_ on C through N_m_. N_x_′−C indicates the direct effect of N_x_ on C after controlling for the indirect effect of N_m_.

^a^ Path value is significant with 95% confidence intervals not overlapping 0.

## Discussion

These results broadly demonstrate that, in a group of cognitively normal older adults, functional connectivity within multiple networks is associated with cognitive performance across multiple domains. In addition, there was evidence of specificity in the relationship between connectivity in the FPCN and executive function, and that the FPCN mediated the relationships of the other networks to cognition. First, we observed significant relationships between connectivity in the DN and FPCN with all cognitive domains and between SN connectivity and processing speed and episodic memory. No significant relationships between DAN connectivity and cognition were observed. Also, we observed a significant relationship between a network-general factor score and a domain-general cognitive factor score. Although these relationships are in broad accordance with previously reported findings (Andrews-Hanna et al., 2007; Wang et al., 2010), the pattern of connectivity to cognition relations in the literature has been inconsistent and few studies have included examination of multiple networks or multiple cognitive domains (see Ferreira and Busatto, 2013, for review). Second, in exploratory analyses of voxelwise relationships with cognition, we observed regional patterns of within-network relationships with cognition suggestive of the possibility that regional coupling at the subnetwork level will be sensitive to associations with variation in cognitive function. Third, we observed specificity only in the hypothesized relationship between connectivity in the FPCN and executive control. Connectivity in no other cortical networks exhibited a specific relationship to any cognitive domain. Fourth, we found that the FPCN had a significant mediating role in nearly all relationships between other networks and cognition. Finally, in all of the above results, we controlled for data quality metrics, including SNR, subject motion, and outlier volumes, to minimize the contribution of potential artifacts to the observed age group differences or to the individual differences among older adults (Power et al., 2012, 2014; Satterthwaite et al., 2012; Van Dijk et al., 2010, 2012).

When relationships among cortical networks were not partialled, the DN and FPCN displayed significant correlations with all cognitive domains, while the SN displayed significant correlations with processing speed and episodic memory. The DAN was not significantly associated with any cognitive domain; this may have been due to its lower correlations with the other networks (Supplementary Table S1), especially if the relationships to cognition operate through interactions between the networks. Alternatively, when using templates specifically derived from the same older adult sample reported here, the DAN template had the lowest spatial correlation (*r*=0.59) with the reference template set (*r* >0.83 for all other networks), suggesting that the DAN may be subject to less robust measurement properties or may undergo more substantial age-related reorganization compared to the other networks. Similar results were previously reported in a subset (*N*=70) of the current sample (Schultz et al., 2014). Along with reports of reorganization of network structure among older adults (Chan et al., 2014), these results raise questions regarding the efficacy of applying templates derived from younger adults to older adult data. Theoretically, because older adults were once young, reference network templates derived from younger adults provide an estimate of baseline organization from which deviations can be measured and compared across individuals in a sample of older adults. Empirically, substituting sample-specific templates for the reference templates in the current data set does not substantially impact any of the reported relationships to cognition. As strategies for deriving network descriptions that capture the idiosyncratic structures and patterns at the individual level continue to develop (Mueller et al., 2013), it will be important to examine how individual variation in network structure and interaction relates to traditional metrics of network integrity and to variation in cognition.

The relatively high correlations between networks (Supplementary Table S1) indicate the potential for a general influence of connectivity on cognition. A network-general factor score was significantly correlated with a domain-general cognitive factor score. These nonspecific relationships confirm a prior study (Andrews-Hanna et al., 2007) examining connectivity between the posterior cingulate and medial prefrontal cortex nodes of the DN that observed relationships of connectivity between these regions with composite measures of processing speed, executive function, and episodic memory in older adults. Until now that basic finding had not been replicated, likely because of large variations in the cognitive measures and connectivity methods employed across studies. That such general correlations are observed across networks and domains supports the varied findings of relationships between different network metrics and study-specific tasks (Damoiseaux et al., 2008; Geerligs et al., 2012; Keller et al., 2015; Mevel et al., 2013; Wang et al., 2010), but additionally suggests that any relationships between a given network and a given cognitive task must be interpreted with an eye toward the likelihood of general relationships across networks and cognitive domains.

A prior report linked connectivity between nodes of the DN, specifically the posterior cingulate and the medial temporal lobe, to memory performance but not to nonmemory tasks (Wang et al., 2010). That result, in addition to our prior theoretical work (Buckner, 2004; Hedden and Gabrieli, 2004), led us to hypothesize a specific link between DN connectivity and episodic memory. However, our results failed to support that hypothesis. In addition to the lack of network-wide results, our voxelwise analysis found no voxels within the parahippocampal region of the DN showing a specific association with episodic memory even at very liberal thresholds. Our connectivity metric examined the link between each voxel and the network-wide timecourse of regions within the DN. It remains possible that measurements targeting associations between medial temporal lobe regions to individual neocortical regions within the DN or specific subnetworks of the DN would exhibit specificity with memory.

In contrast, our results do support the hypothesized specificity of the link between FPCN connectivity and executive function (Hedden and Gabrieli, 2004, 2010; Hedden et al., 2012b). Furthermore, the results of our mediation analyses indicate that the FPCN mediates the relationships of the DN and SN to cognition, as seen by the fact that the indirect path involving the FPCN was significant in all but one case and that, in all cases, the direct path from the DN or SN to cognition was not significant after controlling for the mediating influence of FPCN (Table 2). These results may be interpreted in the context of other findings to suggest that the interconnections of the FPCN with other cortical networks allow the FPCN to exert influence over other networks in service of goal-directed cognitive tasks (Cole et al., 2012, 2013; Spreng et al., 2010, 2013). However, the FPCN also had the largest effect sizes with respect to cognition, and relatively high correlations between the networks (Supplementary Table S1) may result in shared variance of the network-cognition relationships that are assigned to the network with the largest effect size in the models, rather than indicating a causal pathway by which the FPCN mediates or modulates the role of other networks in cognition. Note that our results are based on cross-sectional data; hence, these mediating relationships should be interpreted as indicative of across-person variance rather than within-person variance.

Although our primary results demonstrated network-wide relationships with cognition, we additionally conducted exploratory analyses of the topography of correlations with cognition in each network. These results were suggestive of widespread regional relationships with cognition in the DN and FPCN, with more localized relationships in the SN and DAN. Notably, although the DAN did not exhibit significant relationships with cognition at the network-wide level, there were local regions with potentially meaningful links to each of the cognitive domains. We provide these results for descriptive purposes and to spur hypothesis generation, as we did not have hypotheses regarding regionally specific relationships. In examining these results, it is important to keep in mind that these maps represent only those regions with the largest relationships to cognition in each network.

Because of interest in alterations of across-network relationships during aging (Chan et al., 2014), we conducted exploratory analyses of cluster-level connectivity both within and across networks related to general cognition (see Supplementary Materials and Methods, Supplementary Results, Supplementary Table S2, and Supplementary Fig. S1). Similar to the above, these results were not only suggestive of regional relationships with cognition in the DN and FPCN, but also showed evidence of such relationships in the SN. Across-network relationships were most pronounced between clusters of the DN and FPCN, with evidence of cross-network involvement of frontal nodes of the SN. Relative to the other networks, there was little involvement of the DAN.

Although our study was focused on relationships between network connectivity and cognition among a group of cognitively healthy older adults, our results do not necessarily imply that these relationships are specific to aging within the range of 65–90. The observed correlations between network connectivity and cognitive domains were present after partialling age from all analyses. Furthermore, the results here should be interpreted in the context of our recent finding that when examined in concert with multiple markers of brain health, functional connectivity in the DN and FPCN did not uniquely share age-related variance in cognition (Hedden et al., 2014). Together with those results, the data presented here indicate that the link between network connectivity and cognition is not specifically related to age within the range of 65–90, but rather may become apparent in older adulthood as variability in overall brain integrity becomes altered by multiple pathological cascades prevalent during aging.

Our methodology for measuring connectivity implements a novel framework, TBR (Schultz et al., 2014), which applies an *a priori* network parcellation from a reference dataset to the dataset of interest. This has the advantage of not requiring the current data to define the topography of networks of interest, thus allowing the full power of the dataset to be utilized in probing the relationships between connectivity and cognition. It should be noted that the TBR method does not require use of the specific network templates we chose and is generally applicable to any *a priori* network parcellation scheme. The network templates we used were chosen because they were defined from a large dataset (675 subjects), provided components that clearly represented the four cortical networks of interest for our hypotheses, and were based on data using the identical acquisition protocol as in our study (Schultz et al., 2014). To the extent that other stable network parcellations based on large samples identify these intrinsic networks (Biswal et al., 2010; Smith et al., 2009; Yeo et al., 2011), regardless of minor variations in their measured topographies, we expect that the TBR method would yield similar results in terms of the observed relationships to cognition. However, it remains possible that alternative network parcellations could result in variation in the observed relationships with cognition.

Although we observed a number of correlations with cognition, it should be stressed that all of the significant correlations observed were in the small to moderate range (*r*=0.15–27) (Cohen, 1988). The observed results were significant primarily because our sample size afforded sufficient power to detect effects of this size. Prior findings of relationships between connectivity and cognition may display inconsistencies (see Ferreira and Busatto, 2013, for review), in part, because the typical sample size (50 or smaller) only allows examination of large effects that may be specific to the analytic or sampling methods of a given study. If the true effect size of relationships between network connectivity and cognition is likely to be *r*≈0.25 or less, this implies that detection of such effects with one-tailed α=.05 and power of (1−β)=0.80 requires a sample size of 97 or larger, assuming no multiple comparisons will be conducted [see also (Biswal et al., 2010)]. With the growing availability of large-sample datasets (ADNI, ENIGMA, HCP, OASIS, “1000 functional connectomes,” etc.) and data-sharing agreements, barriers to the use of large-sample studies for examining such relationships are likely to be rapidly removed.

In summary, our data support a general view of network connectivity across multiple cortical networks as being indicative of brain integrity that is linked to cognitive performance across multiple domains in cognitively healthy older adults. In addition, we observed a preferential relationship between FPCN connectivity and executive function, suggesting the potential for more specific relationships. A potential mediating role of the FPCN with respect to the associations of other networks to cognition was suggestive of a central role of the FPCN in how network interactions are linked to cognitive output. Exploratory analyses of regional relationships indicated the possibility of substantial regional variation in how connectivity within and across networks is related to cognition. As we age, functional connectivity in multiple networks may be relevant for understanding variation in cognitive function.

## Supplementary Material

Supplemental data
