# Supplementary material for: Functional Connectivity in Multiple Cortical Networks Is Associated with Performance Across Cognitive Domains in Older Adults
Source: Brain Connect. 2015 Oct 1;5(8):505–16. doi: 10.1089/brain.2014.0327 (PMC4601675; doi:10.1089/brain.2014.0327)
Supplement: Supplemental data [file Supp_Data.pdf]

Supplementary Material

Functional connectivity in multiple cortical networks is associated with performance across cognitive domains in older adults

Emily E. Shaw[1,2], Aaron P. Schultz[1,2,3], Reisa A. Sperling[1,2,3,5], Trey Hedden[1,4]

[1]Athinoula A. Martinos Ctr. for Biomed. Imaging, Dept. of Radiology, Massachusetts Gen. Hosp., Charlestown, MA; [2]Dept. of Psychiatry, [3]Dept. of Neurology, [4]Dept. of Radiology, Massachusetts Gen. Hosp., Harvard Medical School, Boston, MA; [5]Cnt. for Alzheimer Res. and Treatment, Dept. of Neurol., Brigham and Women's Hosp., Harvard Medical School, Boston, MA

Corresponding author: Trey Hedden, Athinoula A. Martinos Center for Biomedical Imaging, 149 13th St., Suite 2301, Charlestown, MA 02129, 617-643-5529, hedden@nmr.mgh.harvard.edu

Supplementary Materials and Methods

Cluster correlation analyses

Using the reference dataset (the 675 participant dataset described in Schultz et al., 2014), each network (shown in Figure 1) was decomposed into functional clusters by selecting the peaks in that template's map and setting the lower threshold at ~60% of that peak's value (chosen to provide complete separation of clusters), then saving the entire cluster associated with that peak as a mask. This resulted in 29 distinct clusters across the networks (6-8 clusters per network). For each older participant in the current dataset, the average timecourse from each cluster was extracted and correlated with the timecourse of every other (within- and across-network) cluster, resulting in 406 cross-cluster correlations. We then computed the partial correlation across subjects of each cross-cluster correlation with the domain-general cognitive score defined above, controlling for age and the quality assessment metrics of SNR, mean movement, and number of outlier volumes. The partial correlations between general cognition and cross-cluster connectivity were plotted in a circular wiring diagram. To correct for multiple comparisons, we examined the cross-cluster correlations using principal component analysis and found 99 components having an eigenvector greater than 1; together these accounted for 94.3% of the variance in the cross-cluster correlations. On this basis, we assumed that the interdependency of the cross-cluster correlations reflects approximately 99 independent tests, and used this as the denominator to achieve a family-wise error corrected $p < .0005$. To further limit the number of comparisons in this exploratory analysis, we do not separately report relationships across the three cognitive domains.

Supplementary Results

Within- and across-network cluster correlations with cognition

The primary analyses examined only within-network correlations with cognition. We further explored simultaneous within-network and across-network connectivity by extracting clusters from the group map in the reference dataset for each network (Supplementary Table 2) and examining relationships between all cross-cluster correlations and domain-general cognition in the older adult dataset. For visualization, these relationships were plotted in a circular wiring diagram (Supplementary Figure 1). Although these results should be taken purely as exploratory, some general impressions can be conveyed. The within-network correlations appeared to be concentrated in the DN, FPCN, and SN. Across-network correlations related to cognition were relatively sparse, but correlations between the right inferior temporal cluster of the FPCN and the posterior cingulate and bilateral angular gyrus clusters in the DN were among the strongest relationships observed. There was also evidence of across-network correlations related to cognition in the SN; however, these were all located in the prefrontal cortex so that anatomic proximity may be an important factor in the observed relationships. Relative to the other networks, there was little involvement of the DAN. To limit the number of comparisons in this exploratory analysis, we do not separately report each cognitive domain.

Supplementary Table 1: Correlations between age, cognition, connectivity, and quality assessment metrics within older adults

1	Age	1	2	3	4	5	6	7	8	9	10
2	Processing Speed	**-0.25**									
3	Executive Function	**-0.25**	**0.85**								
4	Episodic Memory	**-0.32**	**0.56**	**0.70**							
5	DN	**-0.20**	**0.25**	**0.22**	**0.20**						
6	FPCN	**-0.20**	**0.27**	**0.30**	**0.23**	**0.75**					
7	SN	**-0.23**	**0.21**	**0.18**	**0.20**	**0.90**	**0.69**				
8	DAN	**-0.17**	**0.14**	**0.13**	0.07	**0.53**	**0.49**	**0.45**			
9	SNR	**-0.19**	0.09	0.10	0.06	**0.38**	**0.33**	**0.39**	**0.19**		
10	Movement	**0.13**	0.01	0.02	0.02	**-0.30**	**-0.28**	**-0.30**	**-0.16**	**-0.31**	
11	# Outlier Volumes	**-0.14**	**0.15**	**0.14**	0.10	**0.25**	**0.13**	**0.21**	**0.13**	0.06	-0.11

Note. Uncorrected correlations between cognitive factor scores, cortical networks and QA metrics. All networks were significantly correlated with one or more cognitive factor scores. Age and QA metrics also had significant correlations, and were controlled in subsequent analyses. For display purposes, **bold** values indicate significance at $p<0.05$, two-tailed. All variables were included in the primary analyses on an *a priori* basis.

Supplementary Table 2. Cluster-level statistics for regions identified from network templates

Region	Template	x	y	z	F	#Voxels
1. R superior parietal	rFPCN	45	-55	53	252.6	536
2. R inferior temporal	rFPCN	60	-43	-10	170.4	270
3. R dorsolateral prefrontal	rFPCN	48	26	38	199.5	1394
4. R presupplementary motor area	rFPCN	6	29	47	171.1	103
5. L presupplementary motor area	lFPCN	-3	23	50	145.2	97
6. L superior parietal	lFPCN	-33	-64	47	196.0	554
7. L dorsolateral prefrontal	lFPCN	-48	26	23	214.7	926
8. L inferior temporal	lFPCN	-57	-52	-10	188.2	221
9. R frontal eye fields	DAN	27	-4	53	127.0	165
10. R superior parietal	DAN	21	-64	59	180.1	1262
11. R inferior temporal	DAN	51	-61	-10	192.3	325
12. L superior parietal	DAN	-18	-64	56	172.5	899
13. L frontal eye fields	DAN	-24	-4	56	109.8	133
14. L inferior temporal	DAN	-48	-67	-7	195.0	316
15. R middle temporal	DN	63	-10	-22	209.9	388
16. R angular gyrus	DN	51	-61	32	219.9	260
17. R parahippocampus	DN	27	-19	-22	118.2	111
18. posterior cingulate	DN	0	55	32	257.7	489
19. medial prefrontal	DN	0	53	-7	248.3	1716
20. L parahippocampus	DN	-24	-19	-22	124.0	133
21. L middle temporal	DN	-63	-16	-19	221.0	497

22. L angular gyrus	DN	-48	-64	35	252.0	391
23. R insula	SN	36	20	5	229.9	480
24. R middle prefrontal	SN	36	44	29	177.4	405
25. R supramarginal gyrus	SN	60	-34	38	229.4	448
26. anterior cingulate	SN	6	11	47	182.1	284
27. L middle prefrontal	SN	-36	41	32	171.9	275
28. L insula	SN	-33	17	8	221.0	322
29. L supramarginal gyrus	SN	-63	-31	32	201.2	489

Note. Clusters were defined from the network maps in the reference dataset (N=675, Schultz et al., 2014). Coordinates indicate the peak location in the reference dataset. F values indicate the association of each peak to the network template timecourse as derived from the reference dataset. Cluster extents were determined by identifying each peak (maximum F-value) and thresholding the image at ~60% of that peak’s value.

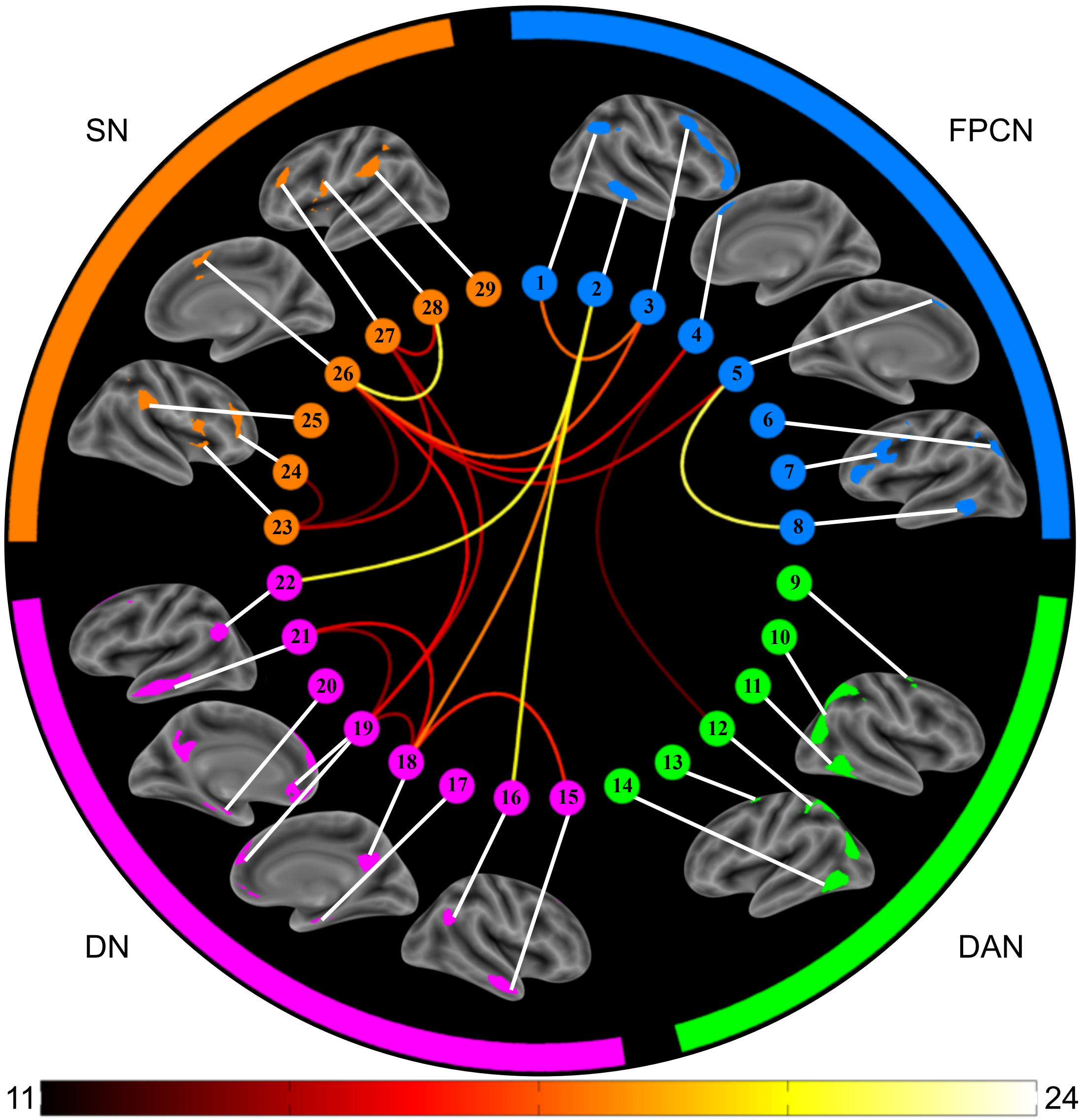

Supplementary Figure 1. Cross-correlations between regional clusters related to general cognition. Circular wiring diagram displays 29 clusters divided into the networks (DN, FPCN, SN, DAN) used to identify each cluster. Nodes are numbered to correspond to the clusters listed in Table 3. Curved lines indicate correlations between nodes that are significantly related to general cognition. The color of the lines, corresponding to the color bar, indicates the strength of the relationship to cognition. Connections showing a relationship to cognition were thresholded at FWE-corrected p<.0005. Lines connecting nodes of the same color indicate connections within a network associated with cognition; lines connecting nodes of different colors indicate connections across networks associated with cognition.
